# A large outbreak of acute gastroenteritis caused by the human norovirus GII.17 strain at a university in Henan Province, China

**DOI:** 10.1186/s40249-017-0236-z

**Published:** 2017-02-01

**Authors:** Xue-Yong Huang, Jia Su, Qian-Chao Lu, Shi-Zheng Li, Jia-Yong Zhao, Meng-Lei Li, Yi Li, Xiao-Jing Shen, Bai-Fan Zhang, Hai-Feng Wang, Yu-Jiao Mu, Shu-Yu Wu, Yan-Hua Du, Li-Cheng Liu, Wei-Jun Chen, John David Klena, Bian-Li Xu

**Affiliations:** 1 Henan Center for Disease Control and Prevention, Zhengzhou, China; 2 Henan Key Laboratory of Pathogenic Microorganisms, Zhengzhou, China; 3 Nanyang City Center for Disease Control and Prevention, Nanyang, China; 4 Program of Global Disease Detection, US Centers for Disease Control and Prevention, Beijing, China; 5 State Key Laboratory of Pathogens and Biosecurity, Institute of Microbiology and Epidemiology, Academy of Military Medical Sciences, Beijing, China; 6 Key Laboratory of Genome Sciences and Information, Beijing Institute of Genomics, Chinese Academy of Sciences, Beijing, China; 7 Division of Global Health Protection, Center for Global Health, Centers for Disease Control and Prevention, Atlanta, USA

**Keywords:** Human norovirus, Acute gastroenteritis outbreak, Epidemiological investigation, Phylogenetic analysis, Henan Province, China

## Abstract

**Background:**

Human noroviruses are a major cause of viral gastroenteritis and are the main etiological agents of acute gastroenteritis outbreaks. An increasing number of outbreaks and sporadic cases of norovirus have been reported in China in recent years. There was a large acute gastroenteritis outbreak at a university in Henan Province, China in the past five years. We want to identify the source, transmission routes of the outbreak by epidemiological investigation and laboratory testing in order to provide the effective control measures.

**Methods:**

The clinical cases were investigated, and analysed by descriptive epidemiological methods according to factors such as time, department, grade and so on. Samples were collected from clinical cases, healthy persons, the environment, water, and food at the university. These samples were tested for potential bacteria and viruses. The samples that tested positive for norovirus were selected for whole genome sequencing and the sequences were then analysed.

**Results:**

From 4 March to 3 April 2015, a total of 753 acute diarrhoea cases were reported at the university; the attack rate was 3.29%. The epidemic curve showed two peaks, with the main peak occurring between 10 and 20 March, accounting for 85.26% of reported cases. The rates of norovirus detection in samples from confirmed cases, people without symptoms, and environmental samples were 32.72%, 17.39%, and 9.17%, respectively. The phylogenetic analysis showed that the norovirus belonged to the genotype GII.17.

**Conclusions:**

This is the largest and most severe outbreak caused by genotype GII.17 norovirus in recent years in China. The GII.17 viruses displayed high epidemic activity and have become a dominant strain in China since the winter of 2014, having replaced the previously dominant GII.4 Sydney 2012 strain.

**Electronic supplementary material:**

The online version of this article (doi:10.1186/s40249-017-0236-z) contains supplementary material, which is available to authorized users.

## Multilingual abstracts

Please see Additional file 1 for translations of the abstract into the five official working languages of the United Nations.

## Background

Human noroviruses are positive-sense single stranded ribonucleic acid (RNA) viruses belonging to the family *Caliciviridae*, and are the most common cause of acute gastroenteritis outbreaks globally [1–3]. The disease burden of noroviruses is substantial and has a significant influence on public health [4, 5]. No vaccines or antiviral therapies are currently available for norovirus infections. Norovirus infections and outbreaks are usually more common in cooler or winter months. Noroviruses are readily transmitted through the fecal-oral route, through person-to-person contact, or through contaminated food or water, meaning that noroviruses spread quickly in enclosed places such as nursing homes, daycare centres, schools, and cruise ships, and are also a major cause of outbreaks in restaurants and catered-meal settings if contaminated food is served [6–8]. Noroviruses have an incubation period of 12–48 hours and symptoms typically include nausea, vomiting, diarrhea, abdominal pain, and fever. Norovirus infections are generally self-limited with mild to moderate symptoms, although severe morbidity and occasional mortality have been observed in immunocompromised patients and the elderly. Symptoms usually last for 1–3 days but can persist longer in young, old, and immunocompromised patients [9–12].

From 4 to 30 March 2015, 753 cases of acute gastroenteritis were reported to the National Notifiable Reportable Diseases Surveillance System (NNDSS) in China from a university in Nanyang, Henan Province. Preliminary investigation indicated that the incident was a large acute gastroenteritis outbreak caused by human norovirus, and the route of transmission might be person-to-person and environmental transmission. We conducted an in-depth epidemiological investigation and laboratory testing in order to identify the source of the outbreak and provide guidance on effective control measures for future outbreaks.

## Methods

### Case definition

The investigated subjects included any person at the university. A clinical case was defined by the onset of diarrhoea ( ≥3 times/day), vomiting ( ≥2 times/day), or diarrhoeawith vomiting (unlimited number of times/day) at the university during the period from 1 March to 3 April 2015. A laboratory-confirmed case was defined when the stool or vomit specimen of a clinical case tested positive for norovirus by real-time reverse transcription polymerase chain reaction (RT-PCR).

### Epidemiological investigation

Medical practitioners reported clinical cases to the Henan Center for Disease Control and Prevention (CDC) from 1 March to 3 April 2015. A questionnaire was used to collect information on demographics, clinical symptoms, date of disease onset, and date of recovery.

### Specimen collection

Medical workers collected a total of 110 stool, vomitus, and/or saliva samples from clinical cases. Additionally, stool samples were collected from students and staff who did not exhibit symptoms of vomiting or diarrhea. Stool samples were collected from 53 people, comprising 23 students and 30 cafeteria food handlers. Additionally, 120 samples of food and water, and from environmental surfaces were collected, including 15 swabs from cafeteria tables, food carts, kitchen cabinets, kitchen rags, and drinking water taps; 41 swabs from doorknobs, classroom tables, toilets, and gargle cups; 50 food samples; and 14 drinking water samples. The school cafeteria provided the food and drinking water samples. All samples were transported frozen to the pathogen laboratory of the Henan CDC.

### Screening for gastroenteritis pathogens

All samples were cultured for bacterial pathogens including Shiga toxin-producing *Escherichia coli*, *Salmonella*, *Shigella*, *Yersinia enterocolitica*, *Vibrio cholerae*, *V. parahaemolyticus*, and *Aeromonas hydrophila*, following the technical procedures of diarrheal pathogenic spectrum surveillance formulated by the China CDC [13]. These samples were also tested for rotavirus, enteric adenovirus, norovirus, sapovirus, and astrovirus using commercially available real-time RT-PCR kits (Shanghai ZJ Bio-Tech Co., Ltd., Shanghai, China or Jiangsu Shuoshi Biological Technology Co., Ltd., Taizhou, China), as per the manufacturer’s protocols [14].

### Full genome sequencing of norovirus

Six samples that tested positive for norovirus (including four stool samples, one vomit sample, and one environmental sample) were randomly selected for whole genome sequencing. Total RNA was directly extracted from the samples using a QIAamp® Viral RNA Mini Kit (Qiagen, Hilden, Germany), according to the manufacturer’s instructions. RNA was eluted in a final volume of 60 μL elution buffer and used immediately or stored at −80 °C. The whole genome sequences of norovirus were amplified by conventional RT-PCR using primers designed in this study (see Additional file 2: Table S1). The RT-PCR products were sent to Sangon Biotech Co., Ltd. (Shanghai, China) for DNA sequencing using an automated ABI 3730 DNA sequencer (Applied Biosystems, Foster City, CA, USA).

### Phylogenetic analysis

The full norovirus genomes were compiled using the SeqMan program in the Lasergene software package (DNASTAR, Version 2.0, Madison, WI, USA). The percentage similarities of nucleotide identity or amino acid identity were calculated using the ClustalX software, [Version 2.0, European Bioinformatics Institute (EMBL-EBI), Cambridge, UK]. Molecular phylogenetic analysis was conducted using the maximum likelihood method based on the Kimura 2-parameter model with MEGA 5 software (available at: http://mega.software.informer.com/5.0/) [15]. The tree with the highest log-likelihood was shown. The percentage of trees in which the associated taxa clustered together was shown next to the branches. Initial tree(s) for the heuristic search were obtained automatically as follows: when the number of common sites was < 100 or < 1/4 of the total number of sites, the maximum parsimony method was used; otherwise the Neighbor-Joining (NJ) method with maximum likelihood (ML) distance matrix was used. The tree was drawn to scale, with branch lengths measured in the number of substitutions per site. Complete norovirus genomes from GenBank were used as a reference, and phylogenetic trees were constructed to type and to understand the molecular epidemiology of the outbreak strain.

### Statistical analysis

All epidemiologic and laboratory data were entered into EpiData 3.1 software (The EpiData Association, http://www.epidata.dk/download.php, Denmark). All statistical analyses were performed using SAS® v9.13 (SAS Institute Inc., Cary, NC, USA). An association of *P* < 0.05 was considered statistically significant.

### Ethical clearance

This research was approved by the Institutional Review Board of the Henan CDC. All participants gave written informed consent for use of their samples for research purposes. Personal identifiable information was stored by the NNDSS, and not provided to any third party for any purpose according to the Law of the People’s Republic of China on the Prevention and Treatment of Infectious Diseases.

## Results

### Descriptive epidemiology

From 4 to 30 March 2015, a total of 753 acute diarrhea cases were reported at a university in Henan Province to the NNDSS in China. The first case, whose main clinical symptoms included diarrhea, nausea, abdominal distension, abdominal pain, and fatigue, without fever or vomiting, occurred in the School of Economics and Management on the third day after the winter holiday. In the next few weeks, a high number of cases with similar clinical symptoms at various schools of the university were reported.

**Fig. 1:**
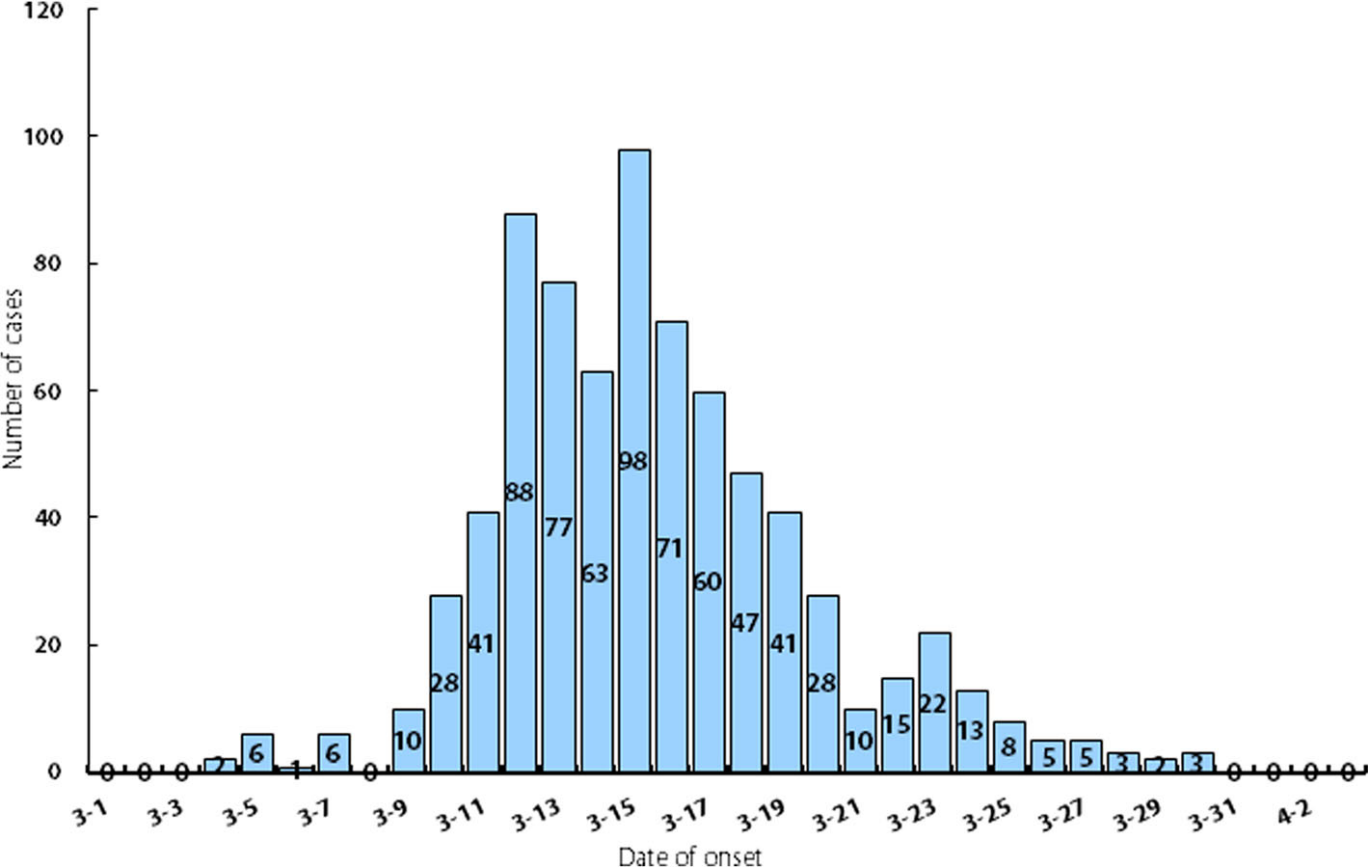
Epidemic curve showing reported cases of acute gastroenteritis by 24 h-intervals at a university in Henan Province, China, 2015

The 753 cases comprised 751 students and two teachers, and the attack rate was 3.29% (753/22 861). Among the cases, 426 were males and 325 were females, with a male–female sex ratio of 1.31. The median age of the cases was 21 years (range: 19–50); the two teachers were 38 years old and 50 years old. The time distribution showed that a main peak of cases occurred (85.26% of the reported cases) between 10 and 20 March (see Fig. 1).

The 753 cases occurred across 16 departments of the university. There was a statistically significant difference with respect to attack rates within the different departments (*χ* ^2^ = 179.92, *P* < 0.001). Two cases were from the Institute of Education, which is a relatively independent unit located on a different campus of the university. The attack rate in grades 1–2 (3.76%) was higher than in grades 3–4 (3.30%), but this was not an important difference (*χ* ^2^ = 3.118, *P* > 0.05) (see Table 1).

**Table 1:** Numbers of reported noroviruses cases by gender, residence, age group and year

Department	No. of students	No. of cases	Attack rate (%)	Gender of cases		Grade of cases	
				Male	Female	1-2	3-4
Electronics School	1 246	51	4.09	31	20	41	10
International Education College	1 418	61	4.30	32	29	31	30
Traditional Chinese Medicine College	1 609	32	1.99	18	14	10	22
Mechanics Institute	1 615	34	2.11	21	13	26	8
Computer and Information Engineering College	896	31	3.46	17	14	26	5
Architecture School	849	43	5.06	23	20	29	14
Economics and Management College	1 415	74	5.23	40	34	29	45
Software School	3 720	114	3.06	68	46	72	42
Biological and Chemical Engineering College	1 092	39	3.57	23	16	20	19
Mathematics and Physics College	419	14	3.34	7	7	8	6
Civil Engineering College	1 381	45	3.26	27	18	39	6
Foreign Languages School	606	30	4.95	13	17	2	28
Humanities and Law College	1 342	74	5.51	41	33	26	48
Art Institute	1 710	76	4.44	42	34	32	44
Music College	599	31	5.18	22	9	12	19
Education College	1 363	2	0.15	1	1	1	1
Total	21 280	751	3.53	426	325	404	347

### Field epidemiologic investigation and preventive controls

On 13 March 2015, both the local CDC and the provincial CDC were notified about an acute gastroenteritis outbreak at a university in Henan Province, and infection control experts initiated a field epidemiologic investigation.

The university is located in southwest Henan in a district with both urban and rural areas, a subtropical to warm-temperate transitional zone, a continental monsoon humid climate, and four distinct seasons. It is a university with 16 departments, including science, engineering, medicine, education, management science, law, economics, and the arts. The university has two separate campuses, the Central Campus and the Education College. At the time of the outbreak, there were 1 581 staff members and 21 280 students, comprising 19 917 students at the Central Campus and 1 363 students at the Education College. Teachers generally do not have meals on campus as they live in an apartment building that is within walking distance of the university. Most students have three meals a day in the university canteens.

A retrospective investigation showed that the first acute gastroenteritis case occurred on 4 March 2015. On 9 March, 10 students with similar clinical symptoms went to see doctors at the university-affiliated hospital. This information was brought to the attention of the hospital administrators who then reported it to the local public health agency. On 10 March, samples of food and drinking water from the university dining hall were collected and tested for multiple enteric pathogens, however, none of the samples tested positive.

A further epidemiological investigation identified clustered cases among roommates and classmates. On 15 March, comprehensive measures were taken to prevent and control this acute gastroenteritis outbreak, including establishing a temporary diarrhea clinic in the school hospital; isolating and treating patients; disinfecting student dormitories, classrooms, and cafeterias with vitalight lamp radiation (Fushan Creator UV & IR Lighting Co., Ltd., Guangdong, China) or 5–6% sodium hypochlorite disinfectant (Dezhou city Sunkang Disinfection Products Co., Ltd., Shandong, China); offering health education; and encouraging enhanced personal hygiene and social distancing. No new cases occurred after 30 March 2015.

### Clinical symptoms

Clinical symptoms were recorded for 471 out of 753 cases. The main symptoms were diarrhea (85.14%), vomiting (65.61%), nausea (69.64%), stomachache (59.45%), abdominal distension (53.29%), and fever (43.77%) (see Table 2). The disease remitted within 72 hours (median: 50 hours, range: 11–72 hours). No cases were hospitalized and there were no deaths.

**Table 2:** Clinical symptoms of 471 clinical cases

Symptom/sign	No. of cases (*n* = 471)	Proportion (%)
Diarrhea	401	85.14
Vomiting	309	65.61
Nausea	328	69.64
Bellyache	280	59.45
Abdominal distension	251	53.29
Fever	206	43.77

### Pathogen detection

From 14 March to 1 April 2015, 110 clinical samples from cases were collected, including 77 stool samples, 24 vomit samples, and nine saliva samples. No bacterial pathogens causing the disease were detected in any samples by culture methods. Thirty-six (32.72%, 36/110) samples from cases tested positive for norovirus using real-time RT-PCR, which comprised 27 stool (35.06%, 27/77), seven vomit (29.17%, 7/24), and two saliva (22.22%, 2/9) samples. Four (5.19%, 4/77) stool samples were positive for rotavirus using real-time RT-PCR. No other gastrointestinal viruses were detected.

### Environmental health investigation

Four (17.39%) of the 23 students without symptoms tested positive for norovirus. All samples from cafeteria food handlers were negative for norovirus. Eleven (9.17%, 11/120) swab samples tested positive for norovirus, which comprised eight toilet and three gargle cup surface samples. The food and drinking water samples were all negative for norovirus and rotavirus. Bacterial pathogens were not detected in the environmental health samples.

### Molecular characterization of norovirus

**Fig. 2:**
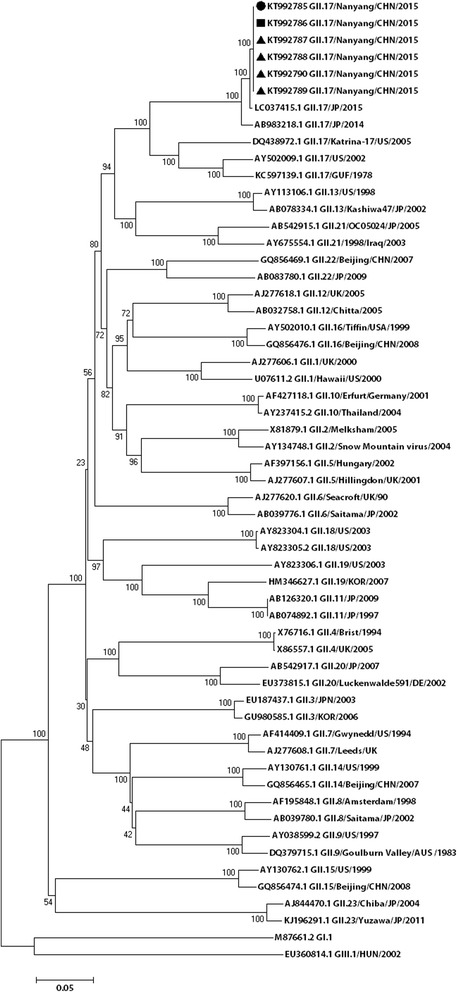
Molecular characterization and phylogenetic analysis of noroviruses, Henan Province

All six strains were typed as GII.17 using the norovirus automated genotyping tool. The result of the phylogenetic analysis, which was performed to verified genotypes of six norovirus strains, coincided with the conclusion above (see Fig. 2).

**Fig. 3:**
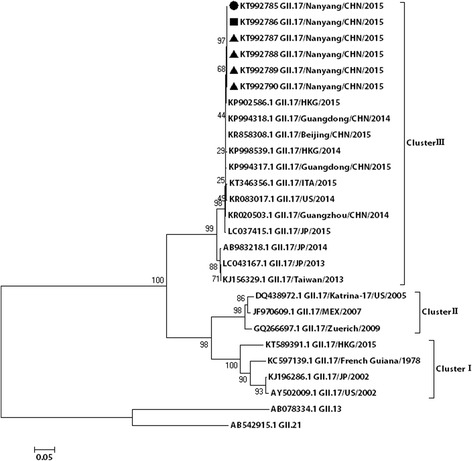
Phylogenetic analyses of noroviruses of the GII.17 strain based on the gene sequence of VP1. The tree shows the comparison between the six noroviruses strains studied and the GII.17 reference strains. The circle represents the stool samples, the square represents the swab samples, and the triangle represents the vomit samples

The complete genomes of the six norovirus-positive samples were determined. Nucleotide sequences were submitted to GenBank (accession Nos. KT992785, KT992786, KT992787, KT992788, KT992789, KT992790). The nucleotide identity among the six GII.17 strains ranged from 95.8% to 99.9%. To further determine the genetic characteristics of the norovirus strains, complete VP1 gene sequences of six norovirus strains from Henan were compared with 21 other GII.17 strains selected from GenBank by phylogenetic analysis. On the basis of VP1 gene sequencing, the GII.17 strains could be divided into three clusters in the phylogenetic tree (I-III): GII.17 strains detected from 1978 to 2002 formed cluster I, GII.17 strains collected from 2005 to 2009 formed cluster II, and cluster III composed of six GII.17 strains from Henan and strains isolated from Hong Kong, Guangdong, Beijing, Italy, Taiwan, Japan, and the USA after 2013 (except for KT589391.1 GII.17/HKG/2015, which belonged to cluster I) (see Fig. 3).

The phylogenetic analysis suggested that viruses from this study clustered with viral sequences obtained from viruses from other provinces in China circulating at a similar time, and co-evolved and co-circulated with those from surrounding provinces.

## Discussion

In this outbreak, a total of 753 acute diarrhea cases were reported at the university; the attack rate was 3.29%. The epidemic curve showed two peaks, with the main peak occurring between 10 March and 20 March, accounting for 85.26% of the reported cases. The statistical analysis identified a significant difference with respect to attack rates among the departments at the university and among grades, and there were obvious clusters among roommates and classmates. All samples collected from the cafeteria food handlers, and food and drinking water samples tested negative for norovirus.

The data suggested that the route of norovirus transmission was more likely to be person-to-person and/or environmental transmission than foodborne or waterborne transmission. Norovirus transmission occurs via the fecal-oral route, usually through ingestion of contaminated food or water, or by direct contact with an infected individual. Environmental transmission occurs when episodes of vomiting or diarrhea contaminate surfaces with infectious virus particles that may persist for weeks [16, 17]. The resilience and persistence of norovirus in the environment allows for its spread through a wide range of common and unexpected sources. It has been estimated that as few as 18 norovirus particles may be sufficient to cause infection in humans [18, 19]. Infected individuals shed a large amount of norovirus in both fecal material and vomitus, contributing to the high number of outbreaks observed annually in environments with close quarters such as cruise ships, restaurants, long-term care facilities, and schools [16, 17, 20]. Dormitories and classrooms are the main places where students study and live, which results in them being highly centralized and relatively confined environments. The swab samples from these environments tested positive for norovirus. After environmental disinfecting measures and hand washing were implemented, the number of cases sharply decreased. Thus, environmental transmission likely contributed to the outbreak [17, 21].

The epidemic curve showed two peaks of incidence. The first peak appeared on the seventh day after the first case occurred, continuing to 20 March. Cases with onset during these 11 days accounted for 85.29% of all reported cases. The incubation period for norovirus is normally 12–48 hours, meaning that this peak period very likely included second- or third-generation transmission. Laboratory testing identified norovirus contamination in environmental samples, not food or water. Epidemiological investigation showed that the first case occurred on the third day of school. According to the incubation period of noroviruses [22, 23], this case was likely infected prior to returning to university after the holidays. The university has students from all over China. From November 2014 through to January 2015, a total of more than 120 identified outbreaks were reported in China. We speculate that the infected student introduced norovirus into the university. The first case was a possible source of transmission, but asymptomatically infected students may also be sources.

Noroviruses can be divided into seven genogroups (GI to GVII) on the basis of sequence differences in the virus VP1 region. GI, GII, and GIV viruses can infect humans. The genogroups are further classified into genotypes with at least nine genotypes belonging to GI, 22 genotypes belonging to GII, and two genotypes belonging to GIV. GII viruses are the most frequently detected (89%), whereas GI viruses cause approximately 11% of all outbreaks [24–27]. During the past decade, most reported norovirus outbreaks were caused by GII.4. New variants of GII.4 have emerged approximately every 2–4 years and have caused norovirus gastroenteritis pandemics globally [28, 29]. Since 1999, the major circulating genotype in mainland China has been GII.4, accounting for 64% of all genotypes detected [30]. GII.17 is another common type of GII viruses. The first GII.17 strain in the National Center for Biotechnology Information databank is from 1978. Since then, GII.17 viruses have sporadically been detected in Africa, Asia, Europe, North America, and South America, and have been circulating in the human population for at least 37 years [31]. In Asia, more widespread circulation of GII.17 was first reported from environmental samples in Korea from 2004 to 2006 [32]. From 2012 to 2013, GII.17 viruses accounted for 76% of all detected norovirus strains in rivers in rural and urban areas in Kenya [33].

A sharp increase in the number of norovirus cases caused by a novel GII.17 virus was observed in Japan during the 2014/15 winter season [34]. This novel GII.17 norovirus was first detected in acute gastroenteritis outbreaks in Guangdong Province in China in November 2014 and thereafter spread rapidly across Asia. From November 2014 through to January 2015, GII.17 norovirus outbreaks were reported in 10 cities of Guangdong Province and represented 83% (24/29) of all outbreaks in Guangdong [35]. During the same winter, there was also an increase in outbreak activity in Jiangsu Province, Zhejiang Province, and other provinces, which could be attributed to the emergence of this novel GII.17 strain [36–38].

Our study is the first report of a genotype GII.17 norovirus causing an outbreak in Henan Province, China. Furthermore, our results indicate that the GII.17 viruses displayed high epidemic activity and have become a dominant strain in China since the winter of 2014, having replaced the previously dominant GII.4 Sydney 2012 strain.

This study had several limitations. First, the early stages of the outbreak did not cause alarm and attention, as the symptoms of the norovirus cases were relatively mild and of short duration. Second, due to a lack of experience with norovirus outbreaks in Henan Province, it took some time before the field epidemiological investigation finally determined that this outbreak was caused by norovirus. Third, there is no routine surveillance data on norovirus infections among infectious viral diarrhea cases in this province, so whether or not GII.17 will continue to circulate is unclear.

## Conclusion

This study identified the largest outbreak of acute gastroenteritis caused by human norovirus GII.17 in Henan Province, China. The environmental transmission contributed to the outbreak, as the students at the university under investigation study and live relatively close in dormitories and classrooms. Environmental disinfecting measures and hand washing should be promoted to prevent such infections and outbreaks because there are not vaccines or antiviral therapies currently available for norovirus infections.

## Additional files

Additional file 1: Multilingual abstracts in the five official working languages of the United Nations. (PDF 733 kb)
Additional file 2: Table S1. Primer names and sequences used to amplify norovirus full genomes. (DOC 36 kb)

## Abbreviations

CDC: Center for Disease Control and Prevention
NNDSS: National Notifiable Disease Reporting System
RNA: Ribonucleic acid
RT-PCR: Reverse transcription polymerase chain reaction
