# Supplementary material for: A large outbreak of acute gastroenteritis caused by the human norovirus GII.17 strain at a university in Henan Province, China
Source: Infect Dis Poverty. 2017 Feb 1;6:6. doi: 10.1186/s40249-017-0236-z (PMC5286658; doi:10.1186/s40249-017-0236-z)
Supplement: Additional file 1: — Multilingual abstracts in the five official working languages of the United Nations. (PDF 733 kb) [file 40249_2017_236_MOESM1_ESM.pdf]

Translation of the abstract into the five official working languages of the United Nations

تفشي كبير لإلتهاب المعدة والأمعاء الحاد الناجم عن سلالة فيروس النورو البشري (GII.17) في جامعة خينان في الصين

شيو يونغ هونغ، جيا سو، تشيان شو لو، تشي تشينغ لي، جيا يونغ تشاو، مينغ لي لي، يي لي، شياو تشينغ شين، باي فان تشانغ، هاي فينغ وانغ، يو تشاو مو، شو يو وا، يان هوا دو، لي تشينغ ليو، وي جان تشين، جون ديفيد كلينا، بيان لي شو

ملخص الدراسة
خلفية الموضوع: تعد فيروسات النورو البشرية هي أحد المسبباب الرئيسية لإلتهاب المعدة والأمعاء الفيروسي وهي من العوامل الأساسية التي أدت الى تفشي التهاب المعدة والأمعاء الحاد. تم الإبلاغ عن أعداد متزايدة و حالات متفرقة لتفشي فيروس النورو في الصين في السنوات الأخيرة. فقد كان هناك عدد كبير لحالات تفشي التهاب المعدة والأمعاء الحاد في إحدى الجامعات في مقاطعة خنان في الصين في السنوات الخمس الماضية. نريد التعرف على مصدر هذا الإلتهاب وطرق العدوى التي أدت الى تفشي هذا الإلتهاب من خلال التحقيق الوبائي والفحوصات المخبرية من أجل تقديم تدابير فعالة لضبط تفشي هذا الإلتهاب.
منهجية البحث: تمت دراسة الحالات السريرة وتحليلها باتباع طرائق البحث الوصفية الوبائية وفقاً لعوامل محددة كعامل الوقت والقسم و المرحلة وغيرها. و تم جمع العينات من الحالات السريرية والأشخاص الأصحاء والبيئة والمياه والطعام في الجامعة. وتم اختبار هذه العينيات من أجل البكتريا والفيروسات المحتمل وجودها. وقد تم اختيار العينات التي ثبت وجود فيروس النورو فيها من أجل تسلسل الجينوم الكامل والذي تم تحليله بعد ذلك.
النتائج: تم الإبلاغ عن 753 لحالات الإسهال الحاد في الجامعة مابين الرابع من شهر مارس والثالث من شهر إبريل عام 2015. وقد أظهر منحنى الوباء قمتين حيث أن فترة القمة الأولى للعدوى كانت مابين العاشر والعشرين من شهر مارس حيث شكلت هذه الفترة 85.26% من الحالات التي تم الإبلاغ عنها. وقد أظهرت معدلات الكشف عن فيروس النورو في العينات من الحالات المؤكدة والناس الذين لم يعانو من أي أعراض و العينات البيئية النسب التالية على التوالي 32.72%، 17.39%، و9.17%. وأظهر تحليل النشوء والتطور أن فيروس النورو ينتمي إلى النمط الجيني GII.17.
الخاتمة: يعد هذا الإندلاع الفيروسي الناجم عن فيروس النورو للتركيب الوراثي GII.17 هو الأكبر والأكثر حدة في السنوات الأخيرة في الصين. فقد أظهر فيروس النورو نشاط وبائي عالي وأصبح السلالة المهيمنة في الصين منذ شتاء 2014 بعد أن حل محل سلالة فيروس GII.4 سيدني التي كانت مهيمنة سابقاً في عام 2012.

Translated from English version into Arabic by Randa82, through

TRANSLATORS WITHOUT BORDERS

人诺如病毒在中国河南一所大学引起的一起急性胃肠炎暴发

黄学勇，苏佳，卢千超，李士正，赵嘉咏，李孟磊，李懿，申晓靖，张白帆，王海峰，穆玉姣，吴蜀豫，杜燕华，刘利成，陈唯君，John David Klena，许汴利

摘要
引言：人诺如病毒是病毒性胃肠炎的主要致病原因，也是引起急性胃肠炎暴发的主要病原。近些年，诺如病毒引起的暴发和散发病例数量在中国不断增长。近期，中国河南一所大学发生了急性胃肠炎暴发。
方法：收集临床病例信息，采用描述性流行病学方法分析病例的时间、院系及年级分布。采集暴发事件发生大学的临床病例、健康人、环境、水及食物标本，对标本进行可能的细菌及病毒学检测。诺如病毒阳性的标本进行全基因组序列测序和分析。
结果：2015 年 3 月 4 日到 4 月 3 日，该大学共报告了 753 例急性腹泻病例，罹患率 3.28%。流行曲线表现双峰，高峰出现在 3 月 10 日到 20 日，85.26%的报告病例发生在期间。确诊病

例、无症状人群和环境标本的诺如病毒检出率分别为 32.72%、17.39%和 9.17%。基因进化分析显示此诺如病毒属于 GII.17 基因型。
结论：此次暴发是近些年 GII.17 基因型诺如病毒在中国引起的最大和最严重的暴发事件。

Translated from English version into Chinese by Xue-Yong Huang and Bian-Li Xu

Une vaste épidémie de gastroentérite aigüe causée par la souche virale du norovirus humain GII.17 dans une université dans la Province du Henan, en Chine.

Xue-Yong Huang, Jia Su, Qian-Chao Lu, Shi-Zheng Li, Jia-Yong Zhao, Meng-Lei Li, Yi Li, Xiao-Jing Shen, Bai-Fan Zhang, Hai-Feng Wang, Yu-Jiao Mu, Shu-Yu Wu, Yan-Hua Du, Li-Cheng Liu, Wei-Jun Chen, John David Klena, Bian-Li Xu

Résumé

Contexte: les norovirus humains sont une cause majeure de gastroentérites virales, et sont les principaux agents étiologiques des épidémies de gastroentérites aigües. Un nombre croissant d'épidémies et de cas sporadiques du norovirus ont été enregistrés en Chine ces dernières années. Une vaste épidémie de gastroentérite aigüe s'est déclarée dans une université de la Province du Hanan, en Chine, au cours des cinq dernières années. Nous voulons identifier la source, et les voies de transmission de l'épidémie par une enquête épidémiologique et des analyses de laboratoire, afin de pouvoir offrir des mesures de contrôle efficaces.
Méthodes: les cas cliniques ont été étudiés, et analysés par des méthodes épidémiologiques descriptives, en fonction de facteurs tels que le moment, le département, la teneur, etc. On a recueilli des échantillons venant de cas cliniques, de personnes en bonne santé, de l'environnement, de l'eau et de la nourriture à l'université. Ces échantillons ont été analysés pour une recherche de bactéries et virus potentiels. Les échantillons testés positifs au norovirus ont été sélectionnés pour un séquençage du génome entier, et les séquences ont été alors analysées.
Résultats: du 4 Mars au 3 Avril 2015, un total de 753 cas de diarrhées aigües ont été enregistrés à l'université ; le taux d'attaque était de 3.29%. La courbe épidémique a révélé deux pics, le pic principal s'étant produit entre le 10 et le 20 Mars, représentant 85.26% des cas enregistrés. Les taux de détection du norovirus dans les échantillons venant de cas confirmés, de personnes n'ayant pas les symptômes, et des échantillons environnementaux, ont été respectivement de 32,72%, 17,39%, et 9,17%. L'analyse phylogénétique a montré que le norovirus appartenait au génotype GII.17.
Conclusions:
C'est la plus vaste et la plus sévère épidémie causée par le norovirus du génotype GII.17 dans ces dernières années en Chine. Les virus GII.17 ont déployé une haute activité épidémique et sont devenus une souche dominante en Chine depuis l'hiver 2014, remplaçant la précédente souche dominante GII.4 de Sydney en 2012.

Translated from English version into French by Ode Laforge, through

TRANSLATORS
WITHOUT BORDERS

Сильная вспышка вызванного норовирусом человека GII.17 острого гастроэнтерита в университете в Китае, в провинции Хэнань

Шу-Йонь-Хуань, Чжа-Су, Цянь-Шао-Лу, Чжи-Жэнь-Ли, Чжа-Йонь-Жао, Мэнь-Лэй-Ли, Йи-Ли, Сяо-Чжинь-Шэнь, Бай-Фань-Чжань, Хай-Фэнь-Вань, Ю-Чжао-Му, Шу-Ю-Ву, Йэнь-Хуа-Дю, Ли-Шэнь-Лю, Уэй-Чжунь-Шэнь, Джон Дэвид Клена, Биань-Ли-Ху

Реферат

История: Норовирусы человека являются главной причиной возникновения вирусного

гастроэнтерита и главными этиологическими агентами вспышек острого гастроэнтерита. За последние годы в Китае было зарегистрирован возросшее число вспышек, а также отдельные случаи заболеваний норовирусной инфекцией. За последние пять лет очень сильная вспышка острого гастроэнтерита произошла в университете, расположенном в китайской провинции Хэнань. Мы хотим установить причины, а также пути передачи этой вспышки заболеваемости с помощью эпидемиологического исследования и лабораторных анализов, направленных на обеспечение эффективных способов контроля.
Методы: Клинические случаи были исследованы и проанализированы методами описательной эпидемиологии на основании таких факторов как время, факультет, курс и т.д. Образцы для анализа были собраны из материала клинических случаев, взяты у здоровых людей, а также были взяты пробы элементов окружающей среды и пробы воды и пищи в университете. Данные образцы были исследованы на возможное наличие бактерий и вирусов. Были отобраны образцы с положительным результатом анализа на норовирусную инфекцию, которые были подвергнуты полному секвенированию генома, после чего все последовательности были проанализированы.
Результаты: В период с 4 марта по 3 апреля 2015 в университете было зарегистрировано, в общей сложности, 753 случая острой диареи; число заболевших составляло 3,29%. Эпидемиологическая кривая показала два пика. Главный пик случился между 10 и 20 марта и составил 85,26% от общего числа зарегистрированных случаев. Частотность выявления норовирусной инфекции в образцах подтвержденных случаев заболевания, у людей без симптомов, а также на основе анализа объектов окружающий среды, составляла 32,72%, 17,39% и 9,17%, соответственно. Филогенетический анализ показал, что данный норовирус принадлежал к генотипу GII.17.
Заключение: Это была самая крупная и самая тяжелая вспышка заболеваемости, вызванная норовирусом генотипа GII.17, за последние несколько лет в Китае. Зимой 2014 года в Китае вирус GII.17 показал высокую эпидемическую активность. До этого самой сильной вспышкой заболевания считалась вспышка 2012 года в Сиднее, вызванная норовирусом GII.4.

Translated from English version into Russian by Natalia Volkova, through

TRANSLATORS WITHOUT BORDERS

Un gran brote de gastroenteritis aguda causado por la cepa GII.17 del norovirus humano en una universidad de la provincia de Henan, China

Xue-Yong Huang, Jia Su, Qian-Chao Lu, Shi-Zheng Li, Jia-Yong Zhao, Meng-Lei Li, Yi Li, Xiao-Jing Shen, Bai-Fan Zhang, Hai-Feng Wang, Yu-Jiao Mu, Shu-Yu Wu, Yan-Hua Du, Li-Cheng Liu, Wei-Jun Chen, John David Klena, Bian-Li Xu

Resumen
Antecedentes: Los norovirus humanos son una importante causa de gastroenteritis viral y los principales agentes etiológicos de los brotes de gastroenteritis aguda. El número de brotes y casos esporádicos de norovirus registrados en China ha crecido en años recientes. En los últimos 5 años se ha dado un gran brote de gastroenteritis aguda en la provincia china de Henan. Pretendemos identificar la fuente y los caminos de transmisión de este brote por medio de una investigación epidemiológica y pruebas de laboratorio con el objetivo de diseñar medidas de control efectivas
Métodos: Se investigaron y analizaron los casos clínicos por métodos epidemiológicos descriptivos de acuerdo con factores tales como la fecha, el departamento, el grado, etc. Se recogieron muestras de casos clínicos, de personas sanas, ambientales, de agua y de alimentos en la universidad. Se analizó la presencia de bacterias y virus en estas muestras. Se seleccionaron las muestras positivas por norovirus para secuenciación del genoma completo y se analizaron las secuencias obtenidas.
Resultados: Del 4 de marzo al 3 de abril de 2015 se registraron un total de 753 casos de diarrea aguda en la universidad, lo que representa una tasa de ataque del 3,29%. La curva epidémica muestra dos picos diferentes, siendo el principal el registrado entre el 10 y el 20 de marzo, que representó el

85,26% del total de los casos. Las tasas de detección del norovirus en las muestras provenientes de casos confirmados, personas sin síntomas, y muestras ambientales fueron 32,72%, 17,39% y 9,17% respectivamente. El análisis filogenético indicó que el norovirus pertenecía al genotipo GII.17.
Conclusiones: Este es el mayor y más grave brote causado por el genotipo GII.17 del norovirus en los últimos años en China. Los virus GII.17 presentan alta actividad epidémica y se han convertido en una de las cepas dominantes en China desde el invierno de 2104, reemplazando a la anteriormente dominante GII.4 Sydney 2012.

Translated from English version into Spanish by Sergio Campo, through

TRANSLATORS
WITHOUT BORDERS
