# Supplementary material for: A large outbreak of acute gastroenteritis caused by the human norovirus GII.17 strain at a university in Henan Province, China
Source: Infect Dis Poverty. 2017 Feb 1;6:6. doi: 10.1186/s40249-017-0236-z (PMC5286658; doi:10.1186/s40249-017-0236-z)
Supplement: Additional file 2: Table S1. — Primer names and sequences used to amplify norovirus full genomes. (DOC 36 kb) [file 40249_2017_236_MOESM2_ESM.doc]

Supplementary Table 1 Primer names and sequences used to amplify norovirus full genomes

| Primer name | Primer sequence (5’ to 3’) | Position |
| --- | --- | --- |
| NV-1F | TGAATGAAGATGGCGTCTAAC | 1-21 |
| NV-533R | TCCAGTAGAGAGACAGCGGT | 514-533 |
| NV-427F | CCACTATGGGCTGTATGTTGA | 427-447 |
| NV-1648R | ATCCCACAGAACCACCCTT | 1630-1648 |
| NV-1557F | CACTGTCAGGCGACCAAAG | 1557-1575 |
| NV-2619R | GAGTCAATAGTGAGGTCATCTTC | 2597-2619 |
| NV-2446F | AGCCATTTACTCTCTACTCCAA | 2446-1467 |
| NV-3599R | TACCCTTGTTGTCACCTCCTT | 3579-3599 |
| NV-3387F | AGATGGGTATGCTTCTCACTG | 3387-3407 |
| NV-4527R | AGTAACCAGTGGGCAATAGAAT | 4506-4527 |
| NV-4393F | GGCACAAATAGTGGCAGAG | 4393-4411 |
| NV-5562R | GGATCATAATTGGTTCAAGAGT | 5541-5562 |
| NV-5395F | GTGGAGTGGAAGTTCAGGTT | 5395-5414 |
| NV-6490R | GCTGATTCCTGATAGAAGTGTTG | 6468-6490 |
| NV-6295F | GACAATGGGAACTACCAAACTA | 6295-6316 |
| NV-7511R | CAATTATTTCTTTTCACTAAACACG | 7487-7511 |
